# Toxicokinetic-Informed Evidential Learning for Applicability-Domain-Aware QSAR/QSPR Prediction of Environmental Contaminant Toxicity

**DOI:** 10.3390/molecules31132203

**Published:** 2026-06-23

**Authors:** Xiankun Huang, Junkai Zheng, Zhihong Zheng, Wenhao Xu

**Affiliations:** 1 Key Laboratory of Groundwater Resources and Environment, Ministry of Education, Jilin University, Changchun 130021, China; xkhuang.ncwu@foxmail.com; 2 College of New Energy and Environment, Jilin University, Changchun 130021, China; 3 College of Water Conservancy and Hydropower Engineering, Hohai University, Nanjing 210098, China; zhengjunkai9500@gmail.com; 4 School of Water Conservancy, North China University of Water Resources and Electric Power, Zhengzhou 450046, China; zzh@ncwu.edu.cn; 5 School of Artificial Intelligence, Beijing University of Posts and Telecommunications, Beijing 100876, China

**Keywords:** QSAR/QSPR, applicability domain, acute-to-chronic extrapolation, toxicokinetic modeling, uncertainty quantification

## Abstract

Quantitative structure–activity relationship and quantitative structure–property relationship (QSAR/QSPR)-based molecular toxicity prediction provides an in silico strategy for prioritizing environmental contaminants when longer-duration bioassay data are sparse. However, many Simplified Molecular-Input Line-Entry System (SMILES)-based machine learning models treat exposure duration as an unconstrained numerical covariate and provide limited information on whether predictions are supported by the observed temporal domain. Here, we evaluated an applicability-domain-aware chemoinformatics framework that combines transformer-derived molecular representations with toxicokinetic-informed temporal encoding and evidential uncertainty estimation. The approach replaces conventional $\log_{10}$-transformed time encoding with a bounded first-order toxicokinetic saturation feature and combines this representation with Deep Evidential Regression to support a joint chemical–temporal view of the QSAR/QSPR applicability domain. Using experimentally derived U.S. EPA Ecotoxicology Knowledgebase (ECOTOX) fish EC50 mortality records, models were trained on 48,728 acute-duration observations and evaluated retrospectively on 2090 temporally separated longer-duration observations. The combined toxicokinetic and evidential model reduced temporal extrapolation error relative to conventional time encoding while maintaining comparable within-domain validation performance. The learned population-level timescale converged to 221 ± 3 h, consistent with accumulation timescales extending beyond standard acute fish test durations. Epistemic uncertainty was positively associated with absolute prediction error across all 10 folds, suggesting that the uncertainty estimates retained sample-level information relevant to applicability-domain-aware molecular toxicity screening. Cross-species analyses further showed that model behavior depended on training time coverage, with greater convergence when available assays covered a larger fraction of the learned timescale. These results suggest that toxicokinetic-informed temporal encoding can improve uncertainty-aware QSAR/QSPR modeling of environmental contaminant toxicity and support prioritization of compounds for further testing, while complementing rather than replacing chronic bioassays.

## 1. Introduction

Quantitative structure–activity relationship (QSAR)-based molecular toxicity prediction provides a chemoinformatics route for prioritizing environmental contaminants when experimental coverage is incomplete. This need is especially evident for longer-duration aquatic toxicity, where standard acute assays such as OECD TG203 (96 h for fish) are widely represented, whereas chronic measurements over weeks to months remain sparse [1,2,3]. Because the number of chemicals in commerce far exceeds the capacity for comprehensive chronic bioassays [4], computational models are increasingly used to support chemical screening and risk-oriented prioritization. In this setting, models must not only predict toxicity from molecular structure but also indicate when longer-duration predictions require extrapolation beyond the temporal domain represented in the training data.

Validated in silico models are also aligned with the 3R principles (replacement, reduction, and refinement) by helping prioritize compounds for follow-up testing rather than expanding long-duration animal assays indiscriminately. For this reason, retrospective validation against large-scale experimental databases is particularly useful: it allows model behavior to be tested across many chemicals and exposure durations without introducing new animal experiments solely for model development. The present study therefore uses temporally separated experimental records from the U.S. EPA Ecotoxicology Knowledgebase (ECOTOX) as a validation setting for acute-to-chronic extrapolation.

Recent advances in molecular representation learning have expanded the scope of QSAR and quantitative structure–property relationship (QSPR) modeling. Transformer-based models can encode Simplified Molecular-Input Line-Entry System (SMILES) strings using pre-trained chemical language models [5,6], and such representations have improved aquatic toxicity prediction across acute and chronic endpoints [7]. More generally, machine learning approaches can complement conventional quantitative structure–activity relationship (QSAR) workflows when trained on sufficiently broad and curated datasets [8,9,10]. Their performance, however, remains sensitive to endpoint definition, molecular coverage, applicability domain, and the extent to which model inputs encode processes that control the measured response [11,12,13].

A common limitation in molecular toxicity models is that exposure duration is usually treated as an ordinary numerical descriptor rather than as a variable constrained by toxicokinetic processes. This representation can be adequate for interpolation within the observed assay window, but it does not encode the saturation behavior expected when uptake and elimination approach steady state. Consequently, long-duration toxicity predictions may rely on unbounded temporal extrapolation, even when the molecular representation itself is learned from large chemical corpora [7,10]. This limitation is important for acute-to-chronic extrapolation because the same chemical structure can be associated with different apparent toxicity values as exposure duration changes.

Three methodological issues therefore arise for chemoinformatics-based toxicity prediction. First, exposure duration is often encoded as an unbounded numerical feature (e.g., $\log_{10}\left(t\right)$), which does not reflect toxicant accumulation under finite elimination rates. Second, point-prediction models do not distinguish predictions supported by the observed temporal domain from those requiring extrapolation. Third, benchmark protocols typically emphasize within-domain validation, whereas temporal extrapolation to longer-duration assays is less frequently examined [7,10]. These issues motivate temporal representations that are not only data-driven but also consistent with basic toxicokinetic constraints.

These issues also broaden the conventional QSAR applicability-domain problem. In standard QSAR methodology, the applicability domain (AD) defines the region of chemical structure, descriptor space, physicochemical properties, or biological response space within which a model is expected to make reliable predictions. It helps distinguish interpolation within the training-supported domain from extrapolation into regions where prediction error may increase. Classical AD assessment is therefore usually based on chemical-space coverage, descriptor leverage, similarity to training compounds, or related distance-based criteria. For duration-dependent toxicity, however, a prediction may be chemically supported while still requiring extrapolation along the exposure-time axis. We therefore use the term chemical–temporal AD as an extension of the standard concept: the chemical component follows the usual QSAR AD definition, whereas the temporal component describes whether the exposure duration is supported by the time range represented during model training. We therefore view acute-to-chronic prediction as a joint chemical–temporal AD problem, in which molecular representation, temporal coverage, and uncertainty estimates must be considered together.

Toxicokinetic–toxicodynamic (TKTD) theory provides a simple mechanistic basis for such a representation. Under first-order kinetics, internal dose accumulation follows a saturation process [14,15,16]:

(1) $$f\left(t,\tau \right)=1-\exp\;\left(-\frac{t}{\tau }\right),$$

where τ is the organism’s chemical elimination time constant. The function is bounded between 0 and 1, reaches half-maximum at t=τln2, and approaches an asymptote as exposure duration increases. This behavior is consistent with the expectation that internal concentrations approach steady state as uptake and elimination balance. Replacing $\log_{10}\left(t\right)$ with a bounded TKTD-based feature may therefore provide a temporal descriptor that is more aligned with longer-duration toxicity prediction. TKTD models are well established in mechanistic ecotoxicology and regulatory risk assessment [14,15,16,17,18], but their use as feature-level inductive biases in deep molecular toxicity models remains less common. Here, TKTD is used in a feature-level sense, whereas established TKTD frameworks include General Unified Threshold models of Survival (GUTS), Dynamic Energy Budget toxicity (DEBtox) models, and TKTD models for primary producers; these model classes explicitly represent toxicokinetic and toxicodynamic state variables and support mechanistic effect assessment rather than simply encoding exposure duration [14,17,18]. The EFSA review emphasizes that these TKTD approaches are mechanistic tools for interpreting exposure–effect relationships, supporting regulatory risk assessment, and extrapolating effects under time-variable exposure when the required biological, kinetic, and endpoint-specific information is available [18]. More broadly, the incorporation of physical equations into neural networks has been explored in climate, weather, and hydrological applications [19,20,21], but toxicokinetic time features have not been systematically evaluated for SMILES-based acute-to-chronic toxicity prediction. Likewise, Deep Evidential Regression (DER) [22] provides a way to estimate epistemic and aleatoric uncertainty in a single forward pass, yet its value for temporal extrapolation in molecular toxicity screening remains unclear.

In this study, we evaluate whether a bounded toxicokinetic temporal encoding can improve uncertainty-aware QSAR modeling of environmental contaminant toxicity. Specifically, we combine ChemBERTa-derived SMILES representations with a learnable TKTD saturation feature and Deep Evidential Regression. We examine the individual and combined effects of the temporal representation and evidential objective, assess the role of domain-informed initialization for learning τ, and test whether training time coverage helps explain model behavior across aquatic taxa. The goal is to evaluate a mechanistically constrained chemoinformatics framework for acute-to-chronic toxicity screening using temporally separated experimental records, with emphasis on molecular structure representation, model validation under temporal extrapolation, and joint chemical–temporal applicability-domain-aware uncertainty estimation.

## 2. Results

### 2.1. Ablation Analysis of TKTD and DER Components

**Figure 1:**
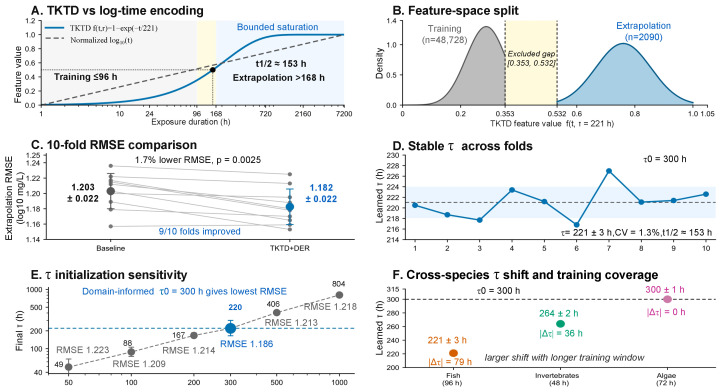
TKTD mechanism, extrapolation performance, and timescale behavior. (**A**) Comparison of conventional log-time and bounded TKTD encoding. (**B**) Separation of training and extrapolation regimes in TKTD feature space. (**C**) Paired 10-fold extrapolation RMSE comparison in Fish EC50. (**D**) Fold-wise stability of the learned time constant. (**E**) Sensitivity of time-constant learning to initialization. (**F**) Cross-species comparison of time-constant shift under matched 5-fold settings.

To examine the separate and combined effects of the TKTD time feature and the DER uncertainty head, we performed a four-condition ablation study using 10-fold SMILES-level cross-validation on Fish EC50 data (Table 1, Figure 1C). Training used records with exposure duration ≤96 h, whereas evaluation used a temporally separated set with duration >168 h (n=2090 per fold).

Replacing $\log_{10}\left(t\right)$ with the TKTD feature while retaining L1 loss did not produce a statistically significant reduction in extrapolation root mean square error (RMSE) (ΔRMSE = −0.007, p=0.171, 6/10 folds improved). Replacing L1 loss with evidential loss while retaining $\log_{10}\left(t\right)$ produced a statistically significant reduction (ΔRMSE = −0.016, p=0.012, 8/10 folds improved). The combined TKTD+DER condition gave the lowest extrapolation error among the four conditions (ΔRMSE = −0.021, p=0.0025, 9/10 folds improved). The combined effect was close to the sum of the individual improvements (−0.007+(−0.016)≈−0.023≈−0.021), which is consistent with partially additive contributions from the two components.

Improvement was specific to the temporal extrapolation domain. Per-fold validation RMSE (t≤96 h) showed no significant difference between conditions (p>0.3), suggesting that the TKTD prior did not degrade interpolation performance. Improvement was confined to the extrapolation domain (t>168 h), consistent with the mechanism: the TKTD feature creates geometric separation in feature space only beyond the training time horizon (Figure 1A,B). The reduction was observed in 9 of 10 folds and occurred only in the longer-duration evaluation set. This pattern is consistent with the intended role of the bounded temporal encoding: within-domain performance remained similar, whereas error decreased when the model was evaluated beyond the training time window. The result therefore suggests that the toxicokinetic time feature reduced error in the setting it was designed to address.

### 2.2. Stability and Biological Plausibility of Learned τ

Across all 10 folds of the TKTD+DER condition, τ converged from its initialization at 300 h to 221±3 h (CV = 1.3%), corresponding to an elimination half-life of τln2≈153 h (approximately 6.4 days; Figure 1D). The low cross-fold variation suggests that the estimated timescale is stable within this dataset and training protocol. This range is also broadly consistent with elimination half-lives reported for moderately lipophilic organic chemicals in fish.

With τ=221 h, the training domain (t≤96 h) maps to TKTD feature values in [0.004,0.353], whereas the extrapolation domain (t>168 h) maps to [0.532,∼1.0] (Figure 1A,B). These intervals do not overlap, which means that the bounded time feature creates a clear geometric separation between the observed and extrapolated duration regimes in feature space.

### 2.3. Sensitivity of τ Convergence to Initialization

To test whether the converged value of τ depended on initialization, we evaluated six initial values spanning two orders of magnitude in five-fold cross-validation (Table 2, Figure 1E). All initializations moved toward shorter timescales, with larger absolute shifts for larger starting values. Two initializations, 200 h and 300 h, converged into a biologically plausible window (final τ=167 and 221 h, respectively), indicating that the model does not require an exact single starting value but does require initialization within an informative timescale range. Across all starting values, extrapolation RMSE remained similar (1.209–1.223, compared with 1.182–1.186 for the 300 h reference runs), showing that predictive accuracy was relatively robust even when the numerical value of τ was not fully identifiable. This behavior is consistent with the analytical form of the gradient:

$$\frac{\partial f}{\partial \tau }=\frac{t}{\tau^{2}}\exp\;\left(-\frac{t}{\tau }\right).$$

This gradient reaches its maximum at t=τ and becomes small when t≪τ or t≫τ. Because the training data are dominated by 96 h records, the gradient signal is expected to be larger when τ lies in a range for which 96 h is a meaningful fraction of the timescale. In the present dataset, that range is approximately 200–400 h, and $\tau_{0}=300$ h falls within this window.

A similar pattern was observed for the learned time constant across ablation conditions. Under the TKTD+DER condition, τ converged to 221 +/−3 h, whereas under TKTD with L1 loss, it converged to 232±3 h (Table 1). This difference suggests that the evidential objective may promote more consistent use of the bounded temporal representation.

### 2.4. Training Time Coverage and Cross-Species τ Convergence

We next examined whether the fish results extended to taxa with shorter standard test durations. Using the matched five-fold configurations across all three taxa, the TKTD+DER model was evaluated on invertebrate and algal data using species-appropriate temporal splits. A descriptive gradient in τ convergence was observed across taxa (Table 2 (Part B), Figure 1F): fish showed the greatest shift from initialization (300→221 h, |Δτ|=79 h), invertebrates showed moderate movement (300→264 h, |Δτ|=36 h), and algae remained unchanged (300→300 h, |Δτ|=0 h).

These results suggest that learning of the TKTD prior may depend in part on the ratio between maximum training duration and the learned timescale, $t_{\mathrm{train},\max}/\tau$. The greatest convergence was observed for fish, which had the highest training coverage ratio among the three taxa, with smaller shifts in invertebrates and algae. This pattern suggests that performance may be more favorable when the available training protocol covers part of the expected toxicokinetic timescale.

### 2.5. Correlation Between Epistemic Uncertainty and Prediction Error

**Figure 2:**
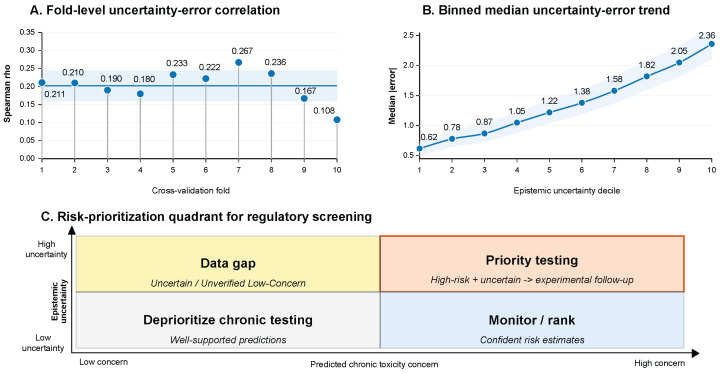
Uncertainty quality under temporal extrapolation. (**A**) Fold-level uncertainty–error correlation on the Fish EC50 extrapolation set. (**B**) Decile-binned trend between epistemic uncertainty and median absolute prediction error. (**C**) Conceptual use of predicted chronic toxicity and epistemic uncertainty to identify predictions for experimental follow-up.

The DER head produced epistemic uncertainty estimates that were positively correlated with absolute prediction error on the Fish EC50 extrapolation set (mean Spearman ρ=0.202±0.042 across 10 folds; all folds p<0.0001; Figure 2A,B). All folds showed positive associations, with individual fold values ranging from 0.108 to 0.267, indicating that higher epistemic uncertainty tended to be associated with larger prediction errors at the sample level. Panel a summarizes the fold-level correlations with the mean and ±1 SD range. Panel b shows the same relationship after decile binning for visualization on the pooled extrapolation set, where median absolute error increases monotonically with epistemic uncertainty.

These results suggest that epistemic uncertainty can support sample ranking. Panel c illustrates that compounds combining high predicted chronic toxicity with high epistemic uncertainty are possible candidates for follow-up, whereas low-toxicity, low-uncertainty predictions can be assigned lower priority. In the matched cross-species comparison, uncertainty quality evidence was directionally consistent with the τ convergence pattern. Fish showed the clearest positive uncertainty–error association, whereas the algal dataset showed near-null correlation, suggesting that the evidential uncertainty signal is more stable when the bounded time feature separates observed and extrapolated duration regimes (Table 2 (Part B); Figure 1F).

### 2.6. Additional Applicability-Domain and Robustness Diagnostics

Sample-level analyses examined chemical-domain membership, longer-duration bias, physicochemical consistency, uncertainty-ranking robustness, and temporal-gap sensitivity. Further details, including sample-level predictions, the data dictionary, and partition-level summary metrics, are provided in the Supplementary Materials. For chemical-domain membership, the fish EC50 mortality dataset contained 52,665 valid records and 3542 unique canonical SMILES. The acute training region (≤96 h) contained 48,728 records and 3472 unique SMILES, the 97–168 h gap zone contained 1847 records and 330 unique SMILES, and the longer-duration extrapolation region (>168 h) contained 2090 records and 487 unique SMILES. Among extrapolation compounds, 430/487 unique SMILES (88.3%) and 2009/2090 records were already represented in the acute training domain. Nearest-neighbor Morgan-fingerprint analysis further showed that the fingerprint-valid extrapolation compounds were generally close to the acute chemical space, with a median maximum Tanimoto similarity of 1.000 and 1988 fingerprint-valid extrapolation records assigned to compounds with maximum Tanimoto similarity ≥0.8. These results support the interpretation that the primary evaluation setting is chemically supported temporal extrapolation, although a small subset still represents simultaneous chemical and temporal extension. Chemical-domain diagnostics for the extrapolation set are summarized in Table 3.

Residual-bias diagnostics showed that the TKTD+DER model was nearly unbiased in the acute training-time region but showed a toxicity-underprediction tendency at longer exposure durations. Using $\mathrm{residual}=\mathrm{observed}\;\log_{10}\left(\mathrm{mg}/L\right)-\mathrm{predicted}\;\log_{10}\left(\mathrm{mg}/L\right)$, the mean residual was −0.029 in the ≤96 h region, −0.589 in the 97–168 h gap zone, and −0.624 in the >168 h extrapolation region. The fraction of records with negative residuals increased from 48.0% in the acute region to 75.1% in the extrapolation region. Within the extrapolation region, the mean residual became more negative from 168–336 h to >672 h, and residuals showed a negative association with $\log_{10}$(duration) (Spearman ρ=−0.112, $p=2.6\times 10^{-7}$; ordinary least-squares (OLS) slope = −0.261 per $\log_{10}$ h). These results show that the model is accurate and nearly unbiased within the acute domain, while longer-duration predictions should be interpreted together with uncertainty estimates. Residual-bias diagnostics across temporal regions and longer-duration bins are reported in Table 4.

Physicochemical stratification did not indicate that model performance was restricted to lipophilic neutral compounds. Across the full dataset, RMSE values were 0.910 for logP < 2, 0.818 for logP 2–4, and 0.921 for logP > 4. In the extrapolation region, the corresponding RMSE values were 1.210, 1.069, and 1.266. Neutral compounds dominated the dataset, whereas charged compounds were sparse and showed higher RMSE and epistemic uncertainty. These results indicate broadly consistent performance across logP strata, while higher uncertainty in charged and high-logP groups reflects weaker data support in sparser chemical subgroups. Physicochemical-stratified performance diagnostics are shown in Table 5.

Structural-domain stratification showed the expected degradation for chemically novel cases, but not a collapse of ranking ability. Exact-identity stratification separated records whose canonical SMILES were present in the acute training domain from records associated with novel canonical SMILES. In the >168 h extrapolation region, seen-chemical records had RMSE = 1.179 and Pearson r=0.665, whereas novel-chemical records had RMSE = 1.536 and Pearson r=0.497. A stricter Bemis–Murcko scaffold split yielded only six novel-scaffold extrapolation records, making that estimate unstable, but it is reported for transparency. The low-similarity subset with maximum Tanimoto similarity <0.4 had RMSE = 2.001 (n=27), confirming that predictions become less reliable in weakly supported chemical space. Structural-domain stratification results are summarized in Table 6.

Uncertainty diagnostics supported the use of epistemic uncertainty as a screening-ranking signal. Across all records, Spearman correlation between epistemic uncertainty and absolute error was 0.396, and the top 10% uncertainty subset had 1.596 times the overall MAE and 3.300 times the bottom-decile MAE. In the extrapolation region, the corresponding uncertainty–error correlation was 0.333, the top-decile MAE was 1.294 times the overall MAE, and the top/bottom MAE ratio was 1.871. Repeated stochastic forward passes gave a mean pairwise Spearman rank correlation of 0.912, mean Kendall correlation of 0.752, and top-20% Jaccard overlap of 0.767 among extrapolation compounds, indicating practical ranking stability. Uncertainty enrichment and ranking-stability diagnostics are reported in Table 7.

Finally, changing the longer-duration threshold from >120 h to >336 h using the trained model did not change the qualitative interpretation. RMSE values remained in the range 1.147–1.274, and the fraction of negative residuals remained 74.7–76.7% across thresholds. This sensitivity analysis supports the robustness of the temporal-gap interpretation, while full retraining under alternative temporal splits remains a useful future extension. The sensitivity analysis for alternative longer-duration thresholds is summarized in Table 8.

## 3. Discussion

### 3.1. Principal Findings and Mechanistic Interpretation

The results suggest that toxicokinetic temporal encoding and uncertainty-aware training contributed differently to SMILES-based molecular toxicity prediction under temporal extrapolation. The TKTD feature provided a bounded representation of exposure time, but this change alone did not produce a statistically significant improvement in longer-duration prediction. Deep Evidential Regression improved temporal extrapolation when used with the conventional time encoding. The greatest gain was observed when the two components were combined, and this pattern was consistent across folds. The fold-wise consistency and restriction to the extrapolation set indicate that the benefit is most relevant when molecular toxicity models are applied beyond well-represented acute assay durations. This result is consistent with the interpretation that the bounded temporal feature changes the geometry of the learning problem, while the evidential objective helps the model use that structure.

The learned value, τ=221±3 h, is interpreted here as a population-level timescale rather than as a compound-specific kinetic parameter. Its low variation across folds suggests that the model repeatedly identifies a similar temporal scale in the training data. This interpretation is consistent with mechanistic TKTD practice but is substantially simpler. GUTS, DEBtox, and primary-producer TKTD models simulate survival, energy-budget allocation, or growth-related effects through explicit kinetic and dynamic processes, whereas the present model uses only the analytical first-order accumulation curve as a temporal inductive bias inside a QSAR/QSPR predictor. Thus, our results complement the EFSA review’s conclusion that full TKTD analyses are valuable for mechanistic and regulatory effect assessment. At the same time, the present QSAR/QSPR framework does not estimate the toxicodynamic parameters or endpoint-specific state variables required by GUTS, DEBtox, or primary-producer TKTD models [14,17,18]. The corresponding elimination half-life is also consistent with values reported for moderately lipophilic organic chemicals in fish. The parameter reflects an aggregate property of a chemically heterogeneous dataset. Its convergence to approximately 2.3 times the maximum training duration (96 h) is consistent with accumulation timescales longer than those captured in acute assays.

Convergence also depended on initialization. The 300 h initialization provided a range in which the available training durations supplied usable gradients. This result suggests that mechanistically constrained parameterization depends both on the form of the governing equation and on whether the data contain enough information to identify the relevant parameter. The gradient analysis ($\partial f/\partial \tau =\left(t/\tau^{2}\right)\exp\left(-t/\tau \right)$) gives a mechanistic explanation: gradient magnitude reaches its maximum when t≈τ, which means that learning is most effective when the training time window overlaps with the timescale being estimated.

The cross-species analysis indicates that τ convergence depends on the temporal coverage of the training data. Fish showed the clearest movement from initialization, whereas invertebrate and algal datasets showed smaller shifts under shorter test protocols. In the present study, the training coverage ratio ($t_{\mathrm{train},\max}/\tau_{\mathrm{final}}$) was 0.43 for fish, 0.18 for invertebrates, and 0.24 for algae, with corresponding relative movement in τ of 26.3%, 12.1%, and 0.08%. Thus, the learned time constant should be interpreted most cautiously when the available training window is short relative to the expected accumulation timescale. The uncertainty analysis clarifies the role of the DER head. Epistemic uncertainty provided a sample-level ranking signal, which was stronger in settings where τ converged well. This is consistent with the view that uncertainty quality partly depends on the upstream representation. The Spearman correlation (ρ=0.202) was positive across all 10 folds, supporting its use as a ranking signal for molecular toxicity screening. The aleatoric component of the DER output was interpreted differently from epistemic uncertainty. In ECOTOX-style retrospective datasets, aleatoric uncertainty reflects irreducible experimental heterogeneity and unmodeled test conditions, including water pH, dissolved organic carbon, hardness, temperature, and inter-laboratory protocol variation. Its increase should therefore be read as a signal of observation noise rather than structural or temporal extrapolation, whereas the epistemic component is the more direct indicator of chemical–temporal applicability-domain support. Chemical-domain analysis showed that most longer-duration records were chemically supported by the acute training domain, so the main evaluation setting is temporal extrapolation with limited simultaneous chemical extension. Residual-bias analysis showed that the model was nearly unbiased within the acute domain but tended to underpredict toxicity at longer durations; this bias was accompanied by uncertainty–error enrichment. Physicochemical stratification further indicated broadly consistent performance across logP strata, while sparse charged and high-logP groups showed higher uncertainty.

### 3.2. Molecular Toxicity Screening Interpretation and Scope

The proposed approach combines three outputs: a longer-duration toxicity estimate, a bounded representation of exposure duration, and a sample-level epistemic uncertainty signal. This combination can support chemoinformatics-based screening by linking QSAR/QSPR prediction with an estimate of prediction reliability and temporal applicability. In this sense, the method extends a static chemical-space AD into a joint chemical–temporal AD: a prediction is considered better supported when both the molecular structure and the exposure duration are represented in a region where the model has learned stable structure–time–effect relationships and assigns low epistemic uncertainty. Compounds predicted to be toxic under longer exposure durations and assigned high epistemic uncertainty may be considered for experimental follow-up, whereas low-toxicity, low-uncertainty predictions can be treated as more reliable within the evaluated domain. This use case complements standard QSAR/ML toxicity screening, which emphasizes chemical descriptors, structural similarity, and statistical validation [8,9,10], and full TKTD or physiologically based pharmacokinetic/physiologically based toxicokinetic (PBPK/PBTK) modeling, which provides stronger mechanistic interpretability but requires substantially richer biological and kinetic input data [18,23,24,25]. The validation strategy is retrospective: the test records are experimentally reported ECOTOX observations separated from the training data by exposure duration. This setting reflects the practical role of in silico toxicity models under the 3R framework, where computational screening can help rank chemicals, define applicability domains, and focus experimental resources.

The results also clarify how the approach should be interpreted. The learned τ represents an effective time constant fitted from discrete toxicity records rather than a mechanistic PBPK or PBTK parameter, and it is shared across chemically heterogeneous samples. Physiologically based toxicokinetic and pharmacokinetic models provide a more detailed mechanistic description by representing organism physiology, compartments, partitioning, clearance, and route-specific exposure; they are especially valuable for in vitro–in vivo extrapolation and chemical-specific risk assessment when the necessary kinetic and physiological information is available [23,24,25]. By contrast, the present approach does not estimate tissue compartments, mass-balance fluxes, or compound-specific kinetic parameters. It is best viewed as a simplified QSAR/QSPR extension that uses a first-order toxicokinetic shape to regularize temporal extrapolation. Because the evaluation focuses on acute-to-chronic extrapolation for compounds represented under shorter exposure durations, τ should be interpreted as a population-level temporal scale rather than evidence of identical toxicokinetic behavior across chemical classes. The physicochemical-stratified analysis supports that performance was not limited to lipophilic neutral compounds, but it does not estimate class-specific τ values. The use of the first-order TK saturation curve also differs from generic bounded statistical transforms such as min–max scaling or sigmoid-normalized time. The TKTD form is the analytical solution of a one-compartment accumulation process, gives the learned parameter a mechanistic interpretation as an effective timescale, and remains bounded as t→∞ without depending on arbitrary training-set endpoints. Future work should examine broader chemical-space validation, compound-class or scaffold-dependent behavior, compound-specific or group-specific τ estimation, cross-species transfer learning, and integration of measured or inferred kinetic information when available.

## 4. Materials and Methods

### 4.1. Dataset and Temporal Split

All experiments used aquatic toxicity records curated from the U.S. EPA ECOTOX Knowledgebase [3,26], a public repository of experimentally reported ecotoxicity tests. Records were processed with RDKit-canonicalized SMILES and $\log_{10}$-transformed toxicity values (mg/L). Canonical SMILES were generated with RDKit MolToSmiles using canonical=True and isomericSmiles=True, so chiral tags and directional bond markers were preserved consistently during tokenization. In the fish EC50 mortality dataset, 326 of 3542 unique SMILES (9.2%) contained at least one stereochemical marker, including 147 with chiral markers and 198 with geometric-isomer markers. The primary dataset comprised fish EC50 mortality records. Cross-species experiments used invertebrate EC50 records (mortality and intoxication effects) and algal EC50 records (population effect). Dataset statistics and temporal split thresholds are summarized in Table 9.

The temporal split protocol ensured clean separation between training and extrapolation regimes. For fish, the training set comprised records with exposure duration ≤96 h (corresponding to the OECD TG203 standard acute test window), and the temporal extrapolation evaluation set comprised records with duration >168 h. Records in the gap zone (97–168 h) were excluded to maintain clear temporal separation. The 97–168 h interval was selected a priori as a buffer between the standard 96 h acute fish assay window and records longer than 7 days. This design reduces ambiguity between interpolation near the acute-test boundary and evaluation under longer-duration temporal extrapolation. Species-specific thresholds were used for invertebrates (train ≤ 48 h, extrap > 96 h) and algae (train ≤ 72 h, extrap > 96 h), reflecting OECD TG211 and ISO 8692 standard [27] protocols, respectively. Data integrity audits confirmed no time-range leakage and no within-fold chemical leakage under SMILES-level splitting. Model selection and hyperparameter comparisons used only acute-duration training/validation folds, whereas longer-duration records were held out for retrospective extrapolation assessment. Duplicate canonical SMILES were assigned consistently within each fold so that the same molecular structure could not appear in both the training and validation partitions of the same within-domain experiment. In the temporal extrapolation set, 88% of unique compounds also appeared in the training set under shorter durations, reflecting the intended evaluation of time-dependent accumulation for known chemical structures rather than structural generalization. The remaining 12% represented simultaneous temporal and chemical-space extension, while the primary validation target remained exposure-duration extrapolation. This design provides a retrospective validation setting based on experimentally reported toxicity records and focuses on evaluating model behavior against observed longer-duration responses. Chemical-domain diagnostics were evaluated for the fish EC50 mortality dataset. Chemical-domain membership was first assessed by canonical-SMILES overlap between the acute training domain (≤96 h) and the longer-duration extrapolation domain (>168 h). We then computed nearest-neighbor chemical similarity using Morgan fingerprints (radius = 2, 2048 bits) and maximum Tanimoto similarity from each extrapolation compound to the acute-domain training compounds. We further stratified sample-level TKTD+DER predictions by exact chemical identity (seen versus novel canonical SMILES), Bemis–Murcko scaffold membership, and a low-similarity subset defined by maximum Tanimoto similarity to the acute training domain. These diagnostics characterized chemical support for the extrapolation set.

### 4.2. Model Architecture

The model builds on the transformer-plus-deep-neural-network (DNN) architecture reported by Gustavsson et al. [7] and comprises (i) a ChemBERTa RoBERTa encoder (6-layer, 768-dimensional CLS token, pre-trained on 10M SMILES) [6], specifically the ChemBERTa-1 PubChem10M masked-language-modeling checkpoint [28] rather than a ChemBERTa-2 multi-task-regression variant; (ii) feature concatenation of CLS embedding, temporal feature, and effect/endpoint one-hot encoding; (iii) a DNN regressor with four hidden layers [2048→1024→512→256], ReLU activations, and dropout = 0.2; and (iv) an output head predicting toxicity values. The DNN was expanded from the reference three-layer configuration [7] to accommodate the four-parameter DER output. All ablation conditions used this architecture, so performance differences reflect the temporal feature and loss function.

### 4.3. TKTD Time Feature

The TKTD time feature replaces $\log_{10}\left(t\right)$:

(2) $$f_{\mathrm{TKTD}}\left(t,\tau \right)=1-\exp\;\left(-\frac{t}{\tau }\right).$$

τ is parameterized as logτ (ensuring τ>0 via exponentiation), initialized at $\tau_{0}=300$ h based on two considerations: (i) the OECD TG203 standard acute test window (96 h) dominates the training signal, and (ii) literature values for moderately lipophilic organic chemicals in fish suggest elimination half-lives in the range of 100–300 h, implying τ≈150–450 h. The chosen initialization lies within this biologically plausible range while being approximately 3× the maximum training duration. This initialization is motivated by the expectation that chronic accumulation occurs on timescales longer than those captured in acute assays, and that the training data (dominated by 96 h records) can provide usable gradient information when τ is initialized near this range. The τ parameter was registered in the AdamW optimizer parameter groups to ensure gradient flow under layer-wise learning rate decay. Sensitivity analysis across six initializations (50, 100, 200, 300, 500, and 1000 h) is reported in Table 2. For the fish dataset, $\tau_{0}=300$ h is approximately 3× the maximum acute-training duration.

### 4.4. Deep Evidential Regression

The DER output head predicts four Normal Inverse-Gamma parameters (γ,ν,α,β), yielding epistemic uncertainty $\sigma_{e}^{2}=\beta /\left[\nu \left(\alpha -1\right)\right]$ and aleatoric uncertainty $\sigma_{a}^{2}=\beta /\left(\alpha -1\right)$. The training loss combines the Normal Inverse-Gamma negative log-likelihood (NIG-NLL) with an evidence regularization term:

(3) $$L_{\mathrm{DER}}=L_{\mathrm{NIG-NLL}}+\lambda \cdot \left|y-\gamma \right|\cdot \left(2\nu +\alpha \right),$$

with regularization weight λ=0.001. The value of λ was selected from a grid search over 0.0001, 0.001, 0.005, 0.010, and 0.050 based on extrapolation RMSE [22].

### 4.5. Training Protocol

Optimization used AdamW [29] with layer-wise learning rate decay (LLRD; decay factor 0.9 per encoder layer) [30], base learning rate $1.5\times 10^{-4}$, OneCycleLR scheduling over 25 epochs, and gradient norm clipping at 1.0. The LLRD strategy applies progressively smaller learning rates to earlier transformer layers while maintaining the base rate for the DNN head and τ parameter, which helps preserve pre-trained representations while allowing task-specific adaptation.

### 4.6. Cross-Validation and Statistical Analysis

Primary experiments (Fish EC50 ablation) used 10-fold SMILES-level K-fold cross-validation (random state = 42). Secondary experiments (cross-species, τ sensitivity) used 5-fold SMILES with the same seed. SMILES-level splitting ensures no molecule appears across training and validation folds within any single experiment. In addition, the longer-duration extrapolation sets were kept temporally separated from the acute-duration training records, so performance estimates reflect retrospective temporal extrapolation rather than random interpolation among similar assay durations. This combination of molecule-level partitioning and duration-level separation was used to evaluate both molecular generalization within folds and the specific acute-to-chronic extrapolation problem addressed by the proposed temporal encoding. The protocol was designed to avoid two common leakage modes in QSAR benchmarking: random splitting of repeated molecular structures across folds and inadvertent use of longer-duration records during model selection.

For the primary comparison, we used a two-sided paired *t*-test on fold-level RMSE values (n=10). Secondary comparisons (n=5) are reported descriptively because of the smaller fold number. We computed the Spearman correlation between per-sample epistemic uncertainty and absolute prediction error on the temporal extrapolation set within each fold independently. Sample-level diagnostic analyses used fish EC50 mortality predictions from the TKTD+DER model. For systematic-bias analysis, residuals were defined as observed $\log_{10}$(mg/L) − predicted $\log_{10}$(mg/L); negative residuals indicate underprediction of toxicity, whereas positive residuals indicate overprediction of toxicity. Physicochemical stratification was performed by logP and formal charge to test whether performance was concentrated in a narrow chemical class. Uncertainty usefulness was assessed by error enrichment in high-epistemic-uncertainty deciles, and ranking stability was examined using repeated stochastic forward passes. Gap-zone sensitivity was evaluated by applying the trained model to alternative longer-duration thresholds. Structural-domain stratification was reported using RMSE, mean absolute error (MAE), Pearson correlation, Spearman correlation, and mean residual for seen-chemical and novel-chemical subsets.

### 4.7. Ablation Study Design

To isolate the contributions of TKTD and DER, we evaluated four conditions in 10-fold cross-validation on Fish EC50 data: (i) Baseline ($\log_{10}\left(t\right)$ + L1 loss); (ii) TKTD-only (TKTD feature + L1 loss); (iii) DER-only ($\log_{10}\left(t\right)$ + DER loss); and (iv) TKTD+DER (TKTD feature + DER loss). All conditions used the same model architecture and differed only in the temporal feature and loss function.

## 5. Conclusions

This study suggests that toxicokinetic temporal encoding and evidential uncertainty training provide complementary information for acute-to-chronic extrapolation in molecular toxicity prediction of environmental contaminants. In fish EC50 data, the combined TKTD+DER model produced the lowest longer-duration extrapolation error among the model variants tested, while within-domain acute validation performance remained similar.

The learned timescale, τ=221±3 h, was stable across folds and is interpreted here as a population-level temporal scale rather than as a compound-specific kinetic parameter. Initialization and training time coverage affected learning of this parameter, suggesting that mechanistically constrained temporal features require attention to the relationship between the available assay duration and the expected toxicokinetic timescale.

Overall, the results suggest that a toxicokinetic temporal prior can constrain longer-duration contaminant toxicity models under temporal extrapolation and can help define a joint chemical–temporal applicability domain for QSAR screening. The approach is most applicable when training records span part of the relevant timescale and should be further evaluated using prospective data, broader chemical-space validation, compound-class analyses, and compound-specific kinetic information where available. The primary fish extrapolation set is largely chemically supported, conclusions are robust to reasonable changes in the longer-duration threshold, and epistemic uncertainty enriches high-error cases. The observed longer-duration toxicity-underprediction tendency reinforces that the model is most appropriate for uncertainty-aware screening and prioritization rather than replacing chronic bioassays.

## Figures and Tables

**Table 1:** Fish EC50 ablation results for TKTD and DER under 10-fold temporal extrapolation evaluation. Values are mean ± SD across folds. $\tau_{\mathrm{final}}$ is reported only for models with the TKTD feature.

Configuration	Val RMSE	Extrap RMSE	Δ vs. BL	*p* Value	$\tau_{\mathrm{final}}$ (h)	Spearman ρ
Baseline ($\log_{10}t$ + L1)	1.094±0.023	1.203±0.022	—	—	—	—
TKTD-only (TKTD + L1)	—	1.196±0.022	−0.6%	0.171	232±3	—
DER-only ($\log_{10}t$ + DER)	—	1.188±0.027	−1.2%	0.012 *	—	0.158±0.067
TKTD+DER	1.074±0.018	1.182±0.022	−1.7%	0.0025 **	221±3	0.202±0.042

Note: * p<0.05; ** p<0.01.

**Table 2:** τ initialization sensitivity in Fish EC50 and cross-species τ convergence under matched 5-fold settings. Values are mean ± SD. |Δτ| denotes the absolute shift from initialization.

*Part A: τ initialization sensitivity (Fish EC50, 5-fold)*						
$\tau_{\mathrm{init}}$ (h)	$\tau_{\mathrm{final}}$ (h)	|Δτ| (h)	Relative shift	Best-val RMSE	Extrap RMSE	Convergence summary
50	49.4±0.1	0.6	1.3%	1.084	1.223	Near initialization
100	87.8±0.7	12.2	12.2%	1.056	1.209	Partial movement
200	167.0±2.0	33.0	16.5%	1.078	1.214	Biologically plausible window
**300 (ref)**	**221.0 ± 2.9**	79.0	26.3%	1.035	1.182	**Biologically plausible window**
500	405.7±3.5	94.3	18.9%	1.081	1.213	Partial movement
1000	804.3±9.1	195.7	19.6%	1.073	1.218	Partial movement
*Part B: Cross-species τ convergence ($\tau_{0}=300$ h, 5-fold)*						
Species (endpoint)	Train ≤	$\tau_{\mathrm{final}}$ (h)	|Δτ| (h)	Relative shift	Coverage ratio	Operational behavior
Fish EC50	96 h	221.0±2.9	78.8	26.3%	0.43	Learned timescale
Invertebrates EC50	48 h	263.6±2.0	36.4	12.1%	0.18	Weakly learned prior
Algae EC50	72 h	299.7±0.6	0.25	0.08%	0.24	Fixed regularizing prior

Note: Italic text identifies the two table parts; bold text highlights the 300 h reference initialization. The table includes column headers for both parts.

**Table 3:** Chemical-domain diagnostics for the fish EC50 temporal extrapolation set. Tanimoto categories are reported for compounds with valid Morgan fingerprints.

Diagnostic Category	Unique SMILES	Records
Seen in acute training domain	430	2009
Unseen in acute training domain	57	81
Maximum Tanimoto ≥0.8	427	1988
Maximum Tanimoto 0.6–0.8	17	20
Maximum Tanimoto <0.6	33	46

**Table 4:** Residual-bias diagnostics across temporal regions and longer-duration bins. RMSE denotes root mean square error, and MAE denotes mean absolute error.

Region or Duration Bin	*n*	RMSE	MAE	Mean Residual	Median Residual	Negative Residuals
Training ≤96 h	48,728	0.854	0.583	−0.029	0.026	48.0%
Gap 97–168 h	1847	1.207	0.885	−0.589	−0.496	73.6%
Extrapolation >168 h	2090	1.190	0.878	−0.624	−0.564	75.1%
168–336 h	860	1.057	-	−0.559	−0.471	72.9%
336–672 h	658	1.153	-	−0.611	−0.533	76.9%
>672 h	572	1.401	-	−0.738	−0.782	76.4%

**Table 5:** Physicochemical-stratified performance diagnostics.

Scope	Stratum	*n*	Unique SMILES	RMSE	MAE	Mean Residual	Mean Epistemic
All records	logP < 2	26,446	1642	0.910	0.644	−0.177	0.362
All records	logP 2–4	15,459	1225	0.818	0.524	−0.002	0.233
All records	logP > 4	9805	602	0.921	0.630	0.090	0.422
All records	neutral	50,793	3401	0.879	0.603	−0.070	0.334
All records	charged	917	68	1.195	0.755	−0.310	0.408
Extrapolation	logP < 2	1233	259	1.210	0.928	−0.686	0.404
Extrapolation	logP 2–4	416	137	1.069	0.691	−0.469	0.192
Extrapolation	logP > 4	405	81	1.266	0.934	−0.622	0.472

**Table 6:** Structural-domain stratification of TKTD+DER sample-level predictions. Residual = observed − predicted $\log_{10}$(mg/L).

Scope	Group	*n*	RMSE	MAE	Pearson *r*	Spearman ρ	Mean Residual
All records	seen-chemical	51,608	0.884	0.605	0.766	0.777	−0.073
All records	novel-chemical	102	1.450	1.002	0.407	0.430	−0.542
Extrapolation > 168 h	seen-chemical	1975	1.179	0.875	0.665	0.670	−0.625
Extrapolation > 168 h	novel-chemical	79	1.536	1.044	0.497	0.476	−0.741
Extrapolation > 168 h	seen-scaffold	2048	1.187	–	0.661	–	–
Extrapolation > 168 h	novel-scaffold	6	2.652	–	0.635	–	–
Extrapolation > 168 h	max Tanimoto <0.4	27	2.001	–	–	–	–

**Table 7:** Uncertainty enrichment and ranking-stability diagnostics.

Metric	All Records	Extrapolation > 168 h
Spearman(uncertainty, absolute error)	0.396	0.333
Top 10% MAE/overall MAE	1.596	1.294
Top-decile/bottom-decile MAE ratio	3.300	1.871
Mean pairwise Spearman under repeated stochastic passes	–	0.912
Mean pairwise Kendall under repeated stochastic passes	–	0.752
Top-20% Jaccard overlap	–	0.767

**Table 8:** Sensitivity analysis for alternative longer-duration thresholds.

Threshold	Gap Width	*n*	RMSE	MAE	Mean Residual	Negative Residuals
>120 h	24 h	3467	1.151	0.857	−0.589	74.7%
>144 h	48 h	3434	1.147	0.855	−0.591	74.9%
>168 h	72 h	2090	1.190	0.878	−0.624	75.1%
>192 h	96 h	2054	1.194	0.880	−0.624	75.0%
>240 h	144 h	1439	1.229	0.911	−0.641	76.0%
>336 h	240 h	1230	1.274	0.955	−0.670	76.7%

**Table 9:** Dataset sizes and temporal split settings for the primary and cross-species experiments. MOR, mortality; ITX, intoxication; POP, population.

Dataset	$n_{\mathrm{train}}$	$n_{\mathrm{extrap}}$	Train ≤	Extrap >	Effect Types
Fish EC50	48,728	2090	96 h	168 h	MOR
Invertebrates EC50	15,234	1456	48 h	96 h	MOR, ITX
Algae EC50	11,203	892	72 h	96 h	POP

## Data Availability

The raw toxicity records used in this study are publicly available from the U.S. Environmental Protection Agency ECOTOX Knowledgebase. The processed data from ECOTOX, temporal split files, model configuration files, trained model weights, code, and analysis scripts supporting this study are available from the Zenodo record at https://zenodo.org/records/20470879 (accessed on 31 May 2026).

## Supplementary Materials

The following supporting information can be downloaded at: https://www.mdpi.com/article/10.3390/molecules31132203/s1, Table S1, Sample-level observed and predicted toxicity values with Deep Evidential Regression uncertainty components; Table S2, A data dictionary describing the variables and units in Table S1; Table S3, Partition-level record counts and error metrics for the acute-training, gap-zone, and temporal-extrapolation subsets; and a README file describing file contents, conventions, and provenance.

## References

[1] van Dijk T.C., Strain O., Hernández-Baquerizo E., de Bruijn J., van den Brink P.J. (2021). The Future of Regulatory Ecotoxicology in a Data-Rich World. Environ. Toxicol. Chem..

[2] European Commission Review of Certain Provisions of Regulation (EC) No 1907/2006 Concerning the Registration, Evaluation, Authorisation and Restriction of Chemicals (REACH), as Laid Down in Its Article 138, 2020. Commission Staff Working Document SWD(2020) 247 Final. https://eur-lex.europa.eu/legal-content/EN/TXT/?uri=CELEX:52020SC0247.

[3] Olker J.H., Elonen C.M., Pilli A., Anderson A., Kinziger B., Erickson S., Skopinski M., Pomplun A., LaLone C.A., Russom C.L. (2022). The ECOTOXicology Knowledgebase: A Curated Database of Ecologically Relevant Toxicity Tests to Support Environmental Research and Risk Assessment. Environ. Toxicol. Chem..

[4] Wang Z., Walker G.W., Muir D.C.G., Nagatani-Yoshida K. (2020). Toward a Global Understanding of Chemical Pollution: A First Comprehensive Analysis of National and Regional Chemical Inventories. Environ. Sci. Technol..

[5] Vaswani A., Shazeer N., Parmar N., Uszkoreit J., Jones L., Gomez A.N., Kaiser L.u., Polosukhin I. (2017). Attention Is All You Need. Proceedings of Advances in Neural Information Processing Systems.

[6] Chithrananda S., Grand G., Ramsundar B. (2020). ChemBERTa: Large-Scale Self-Supervised Pretraining for Molecular Property Prediction. arXiv.

[7] Gustavsson M., Käll S., Svedberg P., Inda-Diaz J.S., Molander S., Coria J., Backhaus T., Kristiansson E. (2024). Transformers Enable Accurate Prediction of Acute and Chronic Chemical Toxicity in Aquatic Organisms. Sci. Adv..

[8] Cherkasov A., Muratov E.N., Fourches D., Varnek A., Baskin I.I., Cronin M., Dearden J., Gramatica P., Martin Y.C., Todeschini R. (2014). QSAR Modeling: Where Have You Been? Where Are You Going To?. J. Med. Chem..

[9] Muratov E.N., Bajorath J., Sheridan R.P., Tetko I.V., Filimonov D., Poroikov V., Oprea T.I., Baskin I.I., Varnek A., Roitberg A. (2020). QSAR without Borders. Chem. Soc. Rev..

[10] Schlender T., Viljanen M., van Rijn J.N., Mohr F., Peijnenburg W.J.G.M., Hoos H.H., Rorije E., Wong A. (2023). The Bigger Fish: A Comparison of Meta-Learning QSAR Models on Low-Resourced Aquatic Toxicity Regression Tasks. Environ. Sci. Technol..

[11] Benfenati E., Manganaro A., Gini G. (2013). VEGA-QSAR: AI inside a platform for predictive toxicology. CEUR Workshop Proc..

[12] Martin T.M., Young D.M. (2001). Prediction of the Acute Toxicity (96-h LC50) of Organic Compounds to the Fathead Minnow (Pimephales promelas) Using a Group Contribution Method. Chem. Res. Toxicol..

[13] Li M., Wei D., Du Y. (2014). Acute toxicity evaluation for quinolone antibiotics and their chlorination disinfection processes. J. Environ. Sci..

[14] Jager T., Zimmer E.I. (2012). Simplified Dynamic Energy Budget Model for Analysing Ecotoxicity Data. Ecol. Model..

[15] Zhao R., Sun D., Fu S., Wang X., Zhao R. (2007). Bioconcentration Kinetics of PCBs in Various Parts of the Lifecycle of the Tadpoles Xenopus laevis. J. Environ. Sci..

[16] Wang L., Liu X., Shan Z., Shi L. (2010). Using electrotopological state indices to model the depuration rates of polychlorinated biphenyls in mussels of Elliptio complanata. J. Environ. Sci..

[17] Ashauer R., Jager T. (2018). Physiological modes of action across species and toxicants: The key to predictive ecotoxicology. Environ. Sci. Process. Impacts.

[18] Ockleford C., Adriaanse P., Berny P., Brock T., Duquesne S., Grilli S., Hernandez-Jerez A.F., Bennekou S.H., Klein M., EFSA PPR Panel (EFSA Panel on Plant Protection Products and their Residues) (2018). Scientific Opinion on the State of the Art of Toxicokinetic/Toxicodynamic (TKTD) Effect Models for Regulatory Risk Assessment of Pesticides for Aquatic Organisms. EFSA J..

[19] Raissi M., Perdikaris P., Karniadakis G.E. (2019). Physics-Informed Neural Networks: A Deep Learning Framework for Solving Forward and Inverse Problems Involving Nonlinear Partial Differential Equations. J. Comput. Phys..

[20] Kashinath K., Mustafa M., Albert A., Wu J., Jiang C., Esmaeilzadeh S., Azizzadenesheli K., Wang R.M., Chattopadhyay A., Singh A. (2021). Physics-informed machine learning: Case studies for weather and climate modelling. Philos. Trans. R. Soc. A.

[21] Willard J., Jia X., Xu S., Steinbach M., Kumar V. (2022). Integrating Scientific Knowledge with Machine Learning for Engineering and Environmental Systems. ACM Comput. Surv..

[22] Amini A., Schwarting W., Soleimany A., Rus D. (2020). Deep Evidential Regression. Adv. Neural Inf. Process. Syst..

[23] Krishnan K., Peyret T., Devillers J. (2009). Physiologically Based Toxicokinetic (PBTK) Modeling in Ecotoxicology. Ecotoxicology Modeling.

[24] Kuepfer L., Niederalt C., Wendl T., Schlender J.F., Willmann S., Lippert J., Block M., Eissing T., Teutonico D. (2016). Applied Concepts in PBPK Modeling: How to Build a PBPK/PD Model. CPT Pharmacomet. Syst. Pharmacol..

[25] Breen M., Ring C.L., Kreutz A., Goldsmith M.R., Wambaugh J.F. (2021). High-Throughput PBTK Models for In Vitro to In Vivo Extrapolation. Expert Opin. Drug Metab. Toxicol..

[26] U.S. Environmental Protection Agency Ecotoxicology (ECOTOX) Knowledgebase Resource Hub. https://www.epa.gov/comptox-tools/ecotoxicology-ecotox-knowledgebase-resource-hub.

[27] (2012). Water Quality—Fresh Water Algal Growth Inhibition Test with Unicellular Green Algae.

[28] Chithrananda S. PubChem10M SMILES BPE 450k ChemBERTa Checkpoint. ChemBERTa-1 Masked-Language-Modeling Checkpoint Trained on PubChem10M SMILES. Hugging Face Model Repository. https://huggingface.co/seyonec/PubChem10M_SMILES_BPE_450k.

[29] Loshchilov I., Hutter F. (2019). Decoupled Weight Decay Regularization. arXiv.

[30] Zhang Y., He P., Gao J. (2020). Rethinking Layer-Wise Learning Rates for Fine-Tuning Pre-Trained Language Models. Proceedings of the 28th International Conference on Computational Linguistics.

